# Comparative Plasma Protein Profiling of Hemoglobin H Disease

**DOI:** 10.1155/2014/340214

**Published:** 2014-06-15

**Authors:** Kamonlak Leecharoenkiat, Wannapa Sornjai, Kornpat Khungwanmaythawee, Atchara Paemanee, Chartchai Chaichana, Sittiruk Roytrakul, Suthat Fucharoen, Saovaros Svasti, Duncan R. Smith

**Affiliations:** ^1^Department of Clinical Microscopy, Faculty of Allied Health Sciences, Chulalongkorn University, 254 Phayathai Road, Bangkok 10330, Thailand; ^2^Molecular Pathology Laboratory, Institute of Molecular Biosciences, Mahidol University, 25/25 Phuttamonthon 4 Road, Salaya, Nakhon Pathom 73170, Thailand; ^3^Proteomics Research Laboratory, Genome Institute, National Science and Technology Development Agency, 111 Thailand Science Park, Phahonyothin Road, Khlong Nueng, Khlong Luang, Pathum Thani 12120, Thailand; ^4^Thalassemia Research Center, Institute of Molecular Biosciences, Mahidol University, 25/25 Phuttamonthon 4 Road, Salaya, Nakhon Pathom 73170, Thailand

## Abstract

HbH and HbH-constant spring (HbH-CS) are the most common forms of *α*-thalassemia detected in the Thai population. The accumulation of excess *β* globin chains in these diseases results in increased red cell hemolysis, and patients with HbH-CS normally have a more severe clinical presentation than patients with HbH disease. This study aimed to detect alterations in the expression of plasma proteins of HbH and HbH-CS patients as compared to normal plasma. Platelet poor plasma was separated from HbH and HbH-CS and normal subjects and differential plasma proteins were detected using two-dimensional gel electrophoresis and identified using LC/MS/MS. A total of 14 differentially expressed proteins were detected of which 5 proteins were upregulated and 9 were downregulated. Most of the differentially expressed proteins are liver secreted proteins involved in hemolysis, oxidative stress response, and hemoglobin degradation. Seven proteins were found to be differentially expressed between HbH and HbH-CS. Levels of haptoglobin, a hemoglobin scavenging protein, were significantly increased in HbH patients as compared to HbH-CS patients. The identification of differentially expressed proteins may lead to a better understanding of the biological events underlying the clinical presentation of HbH and HbH-CS patients and can have application as hemolytic markers or severity predictors.

## 1. Introduction

The *α*-thalassemias are the most prevalent and widely distributed genetic blood disorders of hemoglobin synthesis in Southeast Asia and particularly in Thailand, affecting 16–30% of Thai population. The *α*-thalassemias arise from deletions or mutations affecting one or more *α*-globin genes on chromosome 16, leading to decreased or absent *α*-globin chain production [1, 2]. A single gene deletion (−*α*/*α* *α*) results in *α*-thalassemia silent carrier status and people who carry this state are asymptomatic and present with normal hematologic findings. Two gene deletions (− −/*α* *α* or −*α*/−*α*) cause alpha thalassemia trait (minor) with microcytosis and usually no anemia. The deletion of three genes (− −/−*α*) which leave only one functional *α*-globin gene results in significant production of hemoglobin H (HbH) [3–5]. Four gene deletions (− −/− −) result in significant production of hemoglobin Barts (Hb Barts) that usually results in fatal hydrops fetalis [6, 7].

HbH disease is classified into two main types, deletional and nondeletional HbH disease [8]. Deletional HbH disease commonly results from a deletion removing both *α*-globin genes (*α* ^0^-thalassemia) such as Southeast Asian type (− −^SEA^), Thai (− −^THAI^), or Mediterranean (− −^MED^) on one chromosome 16, plus a deletion removing only a single *α*-globin gene (*α* ^+^-thalassemia) on the other chromosome 16 such as the (−*α* ^3.7^) or (−*α* ^4.2^) deletions [8, 9]. HbH disease in approximately 20% of patients is caused by compound heterozygosity of *α*-thalassemia 1 and *α*-thalassemia 2 resulting from mutation or a small insertion/deletion involving either the *α*2- or *α*1-globin gene on the other chromosome 16. These are collectively labeled as the nondeletional HbH disease and can arise through mutations such as the Constant Spring (CS), Pakse, or Quong Sze mutations [10]. HbH-CS results from the coinheritance of *α* ^0^-thalassemia together with hemoglobin Constant Spring, a structural hemoglobin abnormality caused by a mutation at the termination codon leading to 31 additional amino acid residues attached to the C terminal end of the *α*-globin [11, 12].

The pathophysiology of *α*-thalassemia mainly results from the effect of excess *γ*- and *β*-globin chains resulting from the defective *α*-chain production [13]. In contrast to the excess *α*-globin chains in *β*-thalassemia, the excess *γ*- and *β*-globin chains in *α*-thalassemia do not precipitate in the erythroid precursor cells but form soluble homotetramers which are Hb Barts (*γ*_4_) and HbH (*β*_4_) [14]. These two types of hemoglobin show an extremely high oxygen affinity, no heme-heme interaction, and have poor oxygen carrying ability [15]. Because excess *γ*- and *β*-globin chains form soluble tetramers, the degree of ineffective erythropoiesis in this disease is much less than that seen in *β*-thalassemia [16], and intravascular hemolysis is found to be the major pathophysiology of this disease [14]. It has been proposed that the unstable Hb variants precipitate on the RBC membrane leading to the generation of reactive oxygen species that induce hemolysis resulting in the release of hemoglobin to the circulation. Patients with HbH-CS disease have an additional mechanism for hemolysis because the Constant Spring variant chains damage the red-cell membrane directly, causing an increased influx of water that is followed by hemolysis [17]. The alpha globin variant Constant Spring chains also form tetramers with *β*-globin chains, creating hemoglobin Constant Spring, thereby reducing the number of free *β*-globin chains available to form *β*-globin tetramers of HbH.

Clinical presentation among HbH patients is heterogeneous based on the underlying genotype. Patients with deletional HbH disease (− −/−*α*) usually have an intermediate clinical presentation [9]. In a steady state, most patients have no obvious clinical symptoms but after a complication has occurred the hemoglobin level can occasionally drop significantly. Such a condition can occur during a febrile disease or due to exposure to oxidant chemicals, causing increased hemolysis [18]. Previous studies have shown that compound heterozygosity of HbH with *α*-globin variants such as HbH-CS has a more severe clinical presentation, and HbH-CS produces a disease with hemolytic anemia, jaundice, and splenomegaly suggesting that accumulation of oxidized mutant *α*-globin has a hemolytic effect.

Plasma is the fluid that circulates and surrounds all tissues and organs. It is the most popular and well-accepted choice for clinical testing, and a large number of plasma proteins originate from either normal and abnormal cells or tissues. Alterations in the expression of specific plasma proteins have been found to have association with disease processes [19]. However, plasma contains a number of high abundance proteins including albumin and immunoglobulins which can mask the expression of proteins present in the plasma at medium or low abundance [19]. Specific depletion of these high abundance proteins is therefore commonly employed in analysis of the serum proteome of disease states and this strategy was used to investigate plasma proteins in patients with both deletional and nondeletional HbH disease compared to normal controls.

## 2. Materials and Methods

### 2.1. Sample Collection

A total of 12 HbH, 10 HbH-CS patients, and 12 normal controls were included in this study. The protocol was approved by the Committee for Human Rights related to Experimentation, Mahidol University. Approximately 5 mL of patient and normal control peripheral blood was collected after having ethical approval and individual informed consent. Diagnosis of thalassemia was determined after analysis using HPLC (Variant Hemoglobin Testing System, Bio-Rad, Hercules, CA) for Hb typing and multiplex Gap-PCR as described previously [20, 21]. Normal controls were screened to be free of any thalassemia or hemoglobinopathies by RBC indices, Hb typing, multiplex Gap-PCR, and allele specific PCR [22]. Complete blood counts and RBC indices of both normal subjects and thalassemia patients were determined using an automated cell counter (ADVIA210, Bayer, Tarrytown, NY, USA).

### 2.2. Purification of Plasma Protein

To prepare plasma proteins for 2D analysis, platelet-poor plasma was separated by centrifugation of EDTA treated whole blood for 10 min at 3000 rpm twice and samples were kept at −80°C until use. High abundant plasma albumin and IgG were depleted from pooled samples (6 normal controls, 6 HbH, or 4 HbH-CS) using the Thermo Scientific Pierce Albumin/IgG Removal Kit (Pierce Biotechnology, USA), according to the manufacturer's protocol. Proteins were precipitated from the final eluate with 600 *μ*L methanol, 150 *μ*L chloroform, and 450 *μ*L distilled water and after centrifugation at 20,000 ×g for 5 minutes pellets were washed with 1 mL of methanol followed by centrifugation at 20,000 ×g for 5 minutes. The protein pellets were resolubilized in lysis buffer containing 7 M urea, 2 M thiourea, 4% w/v CHAPS, 100 mM dithiothreitol (DTT), and 1% protease inhibitor cocktail (Bio Basic Inc., Markham, Ontario, Canada). Concentration of the purified plasma proteins was measured by the Bradford assay (Bio-Rad, Hercules, CA) with BSA as a standard.

### 2.3. Two-Dimensional Gel Electrophoresis of Plasma Protein

2D gel analysis was carried out as described previously [23]. Briefly, 100 *μ*g of purified protein was loaded onto Immobiline Drystrips (pH 3–10 NL, 7 cm) containing 2% IPG buffer (Amersham Biosciences) and 0.5% bromophenol blue and the strips were rehydrated for 12 hrs. Proteins were subjected to isoelectric focusing with a multiphor II electrophoresis system (Amersham Biosciences) at the following voltages 300 V for 200 Vh, 1000 V for 300 Vh, 3000 V for 4000 Vh, 5000 V for 4500 Vh, and 5000 V for 3000 Vh. After focusing, the IPG strips were reduced in equilibration buffer (50 mM Tris-HCl (pH 8.8), 6 M urea, 30% v/v glycerol, 2% SDS w/v, and 1% bromophenol blue) supplemented with 100 mM DTT for 15 min and then alkylated in the equilibration buffer containing 150 mM iodoacetamide (IAA) for 30 minutes. The proteins were separated in the second dimension via 12.5% SDS-PAGE. Each pooled sample was run on duplicate 2D gels. Gels were subsequently stained with 0.1% Coomassie Brilliant Blue G250 in 40% methanol for 48 hrs and destained with milliQ water for 6 hrs. The stained 2D gels were scanned under visible light at 300 *μ*m/pixel resolution. Image data was analyzed using ImageMaster 2D Platinum version 7.0 software (Amersham Biosciences). Statistical analysis was performed by Student's *t*-test with a *P* value of less than 0.05 and more than 1.5-fold intensity was considered as statistically significant.

### 2.4. Tryptic in Gel Digestion and Protein Identification by LC/MS/MS

Differentially expressed protein spots were cut from the stained gels and subjected to in-gel tryptic digestion according to the method described elsewhere [24]. Peptide mixtures were analyzed by ultra-performance liquid chromatography (UPLC) (Ultimate 3000, Dionex, USA) coupled to a micrOTOF-Q II ESI-Qq-TOF mass spectrometer (Bruker Daltonics, Germany). The MS/MS spectra produced from each sample were searched against the NCBI databases using the MASCOT search engine (Matrix Science, London, UK). Function, tissue specificity, and involvement in disease of the identified plasma proteins were determined using the UniProt database (http://www.ebi.uniprot.org/).

### 2.5. Western Blot Analysis

Western blotting was carried out essentially as described previously [23]. A total of 30 *μ*g of protein were separated by 12% SDS-PAGE and proteins subsequently were transferred to 0.2 *μ*M nitrocellulose membranes by a wet transfer system. The membranes were blocked with 5% nonfat skim milk in 1x TBS for 1 hr. After blocking, the membranes were incubated with either a 1 : 3000 dilution of a mouse monoclonal anti-human haptoglobin antibody (sc-365396, Santa Cruz Biotechnology Inc., Santa Cruz, CA) for 2 hrs and subsequently incubated with a 1 : 8000 dilution of a rabbit anti-mouse IgG secondary antibody conjugated with horseradish peroxidase (A9044, Sigma, Sigma-Aldrich, St Louis, MO) for 1 hr or with a 1 : 8000 dilution of a polyclonal rabbit anti-human apolipoprotein A-I antibody (sc-30089, Santa Cruz Biotechnology Inc.) for 2 hrs followed by a 1 : 4000 dilution of a HRP-conjugated goat anti-rabbit IgG polyclonal antibody (Pierce Biotechnology, USA) for 1 hr. Protein expression signal was developed using the Enhanced Chemiluminescence Plus system (Amersham Biosciences).

### 2.6. Statistical Analysis

Protein band intensities from western blot analyses were quantitated using the Quantity One 1D analysis software v.4.6.0 (Bio-Rad). Results are presented as the mean value together with the standard error of the mean (SEM) calculated by the GraphPad Prism 5 software (GraphPad Software, La Jolla, CA). Statistical analysis was performed using the Student's *t*-test with *P* < 0.05 considered to be significant.

## 3. Results

### 3.1. Clinical Data of Normal Control, HbH, and HbH-CS

Clinical data including complete blood count (CBC) indices of 12 normal controls, 12 HbH, and 10 HbH-CS are summarized in Table 1. HbH and HbH-CS patients had significantly lower levels of Hb, Hct, MCV, MCH, and MCHC as compared to normal controls but did not show significant differences in WBC and RBC counts.

### 3.2. Comparative Plasma Protein of HbH and HbH-CS by 2D Gel Electrophoresis

**Figure 1:**
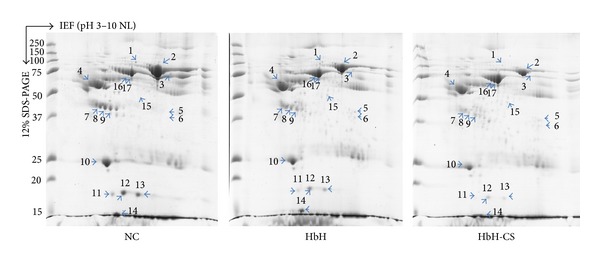
Differential expression of plasma proteins comparing between normal control and HbH disease. Three representative 2D gels of normal control, HbH, and HbH-CS plasma proteins are shown. The protein spots were analyzed using Image Master 2D-Platinum version.5 software (Amersham Biosciences). Comparing between normal control and HbH patients, a total of 17 significantly different protein spots with 11 downregulated proteins (spot numbers H01, H02, H03, H07, H08, H09, H10, H11, H12, H13, and H14) and 6 upregulated proteins (spot number H04, H05, H06, H15, H16, and H17) were detected. Ten differential plasma protein spots were detected when compared between HbH and HbH-CS including 8 downregulated (spot number H03, H07, H08, H09, H11, H12, H13, and H14) and 2 upregulated (spot number H16 and H17).

To compare the expression of plasma proteins between deletional (HbH) or nondeletional (HbH-CS) and normal controls, platelet-poor plasma was further depleted for plasma albumin and IgG and the remaining proteins were analyzed on duplicate 2D gels. A total of 17 spots were detected by spot matching and quantitative intensity analysis as being differentially expressed between the samples (Figure 1). Of these, 11 spots were downregulated in HbH cases (both deletional and nondeletional) and 6 spots were upregulated. The differentially expressed spots were excised from the gels and subjected to identification by in gel tryptic digestion and LC/MS/MS analysis. Results showed that the 17 spots represented 14 different proteins (Table 2), of which 5 were upregulated. Based on protein function analysis, the majority of these proteins (9/14) are secreted from liver cells. Both IgG chains (spots H05, H06, and H15) and serum albumin (H16 and H17) were detected as being differentially upregulated between HbH samples and normal controls, but, as these were specifically depleted from the samples prior to 2D electrophoresis, the result is of unclear significance. Haptoglobin (spots H11, H12, and H13) and haptoglobin-related protein (spot H09) were both downregulated in patients, as were apolipoprotein A-1 (spot H10) and the oxidative stress response proteins transthyretin (spot H14) and serum paraoxonase (spot H07).

Analysis of the differences between deletional HbH and nondeletional HbH-CS revealed 10 protein spots representing 7 different proteins showing differential expression (Table 3). This included 6 proteins that were downregulated in HbH-CS as compared to deletional HbH, namely, complement C3 (spot H03), haptoglobin (spots H11, H12, and H13) and haptoglobin-related protein (H09), the oxidative stress response proteins transthyretin (spot H14), serum paraoxonase (spot H07), and actin (spot H08). Serum albumin (spots H16 and H17) was detected as upregulated in HbH-CS as compared to HbH, but again this is of uncertain significance.

### 3.3. Validation of 2D Expression Profile

**Figure 2:**
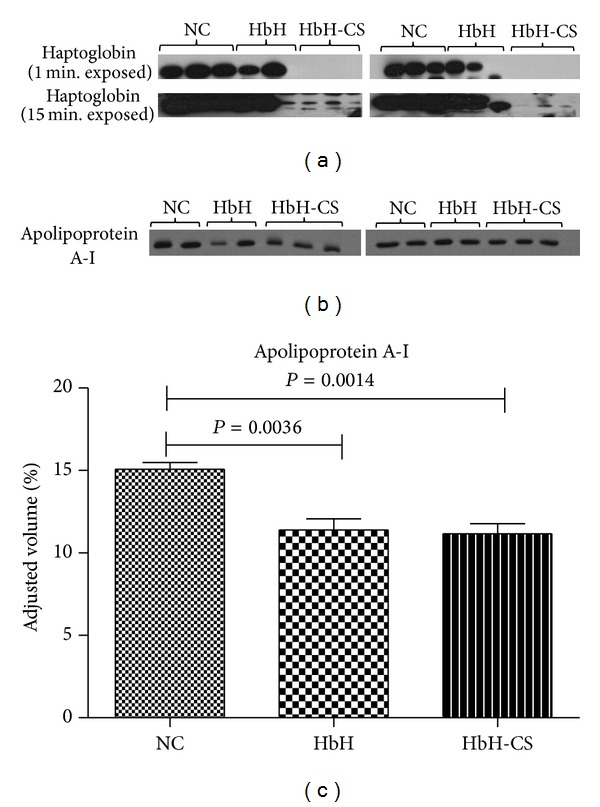
Western blot analysis of plasma haptoglobin and apolipoprotein A-I. (a) Expression of haptoglobin was validated in 6 normal controls, 6 HbH, and 6 HbH-CS. The decrease expression of haptoglobin in HbH disease compared to normal control and HbH-CS compared to HbH disease was detected. (b) Validation of apolipoprotein A-I was done in 4 normal controls, 4 HbH, and 6 HbH-CS. (c) Quantitative analysis of data from (b).

To confirm the differential protein expression profile observed in the 2D analysis, two proteins were selected for evaluation by western blotting. One protein, apolipoprotein A-1, was selected as being differentially regulated between HbH disease (deletional and nondeletional) and normal control (but not between HbH and HbH-CS), while the second protein was differentially regulated between HbH disease (deletional and nondeletional) and normal control and between deletional HbH and nondeletional HbH-CS patients. For validation a second independent cohort of 6 HbH patients, 6 HbH-CS patients, and 6 normal controls was recruited. Consistent with the data from the 2D gel analysis, a significant downregulation in the expression of haptoglobin and apolipoprotein A-I was seen in HbH and HbH-CS as compared to normal control (Figure 2) and significantly HbH-CS showed a lower expression level of haptoglobin than HbH, while apolipoprotein A-1 did not, which was consistent with the proteomic analysis results.

## 4. Discussion

The pathophysiology of HbH disease results from imbalanced globin chain production [8], whereby the reduced *α*-globin chain synthesis results in precipitation of excess *β*- or *γ*-globin chains in RBC membrane [9] and eventual hemolysis [25]. We recently published a proteomic analysis of differential protein expression of cultured erythroblasts from HbH-CS patients as compared to normal controls [26], but to date there is no published information on differences in the global expression of plasma proteins in HbH disease. This study compared the expression level of plasma proteins between normal control and HbH disease and investigated both deletional HbH disease and nondeletional HbH-CS disease. Seventeen differentially expressed spots representing 14 different proteins were detected, some of which, including haptoglobin, complement C3, apolipoprotein A-I, serotransferrin, albumin, and immunoglobulin, have been previously identified as differentially expressed in thalassemia disease [27–30]. Two of these proteins, albumin and immunoglobulin, were specifically depleted prior to analysis as they are abundant proteins that can mask differential expression of less abundant proteins, and as such the altered levels of these proteins seen in this study remain of uncertain significance. The identification of haptoglobin, haptoglobin-related protein, and complement C3 as being differentially regulated is consistent with the known pathology of HbH disease. Haptoglobin and haptoglobin-regulated protein are hemoglobin scavenging proteins [31]. After intravascular RBC hemolysis, hemoglobin is released to the plasma, where it dimerizes and is subsequently rapidly bound by the serum protein haptoglobin. The haptoglobin-hemoglobin complex exposes a neoepitope that is recognized by the hemoglobin scavenger receptor, CD163, expressed on the surface of monocytes/macrophages and these cells mediate haptoglobin-hemoglobin endocytosis and degradation [32]. This study showed decreased levels of plasma haptoglobin in HbH disease most likely because haptoglobin is degraded and not recycled as part of the reticuloendothelial scavenging system [33]. Decreased levels of haptoglobin were detected in HbH-CS as compared to HbH disease, which suggests that levels of haptoglobin correlate with the severity of HbH disease and could serve as an independent severity predictor in patients. Apolipoprotein A-1 was found to be downregulated in both deletional and nondeletional HbH as compared to normal controls, although there was no apparent difference between the levels in deletional and nondeletional HbH, in contrast to the result seen for haptoglobin. Apolipoprotein A-1 has been detected as part of the hemoglobin interactome [34], and apolipoprotein A-1 complexes together with hemoglobin and haptoglobin have been suggested, with apolipoprotein A-1 acting as an antioxidant to decrease the redox activity of hemoglobin [34]. Additionally, this study found that the level of complement C3 in HbH patients was decreased as compared to normal control. This is consistent with data reported in *β*-thalassemia/HbE patients [27] and might be due to oxidation of protein band 3 on RBC membranes which can activate complement-mediated phagocytosis by macrophages, leading to phagocytosis of the damage RBC.

A recent analysis of the serum proteome of mild and severe *β*-thalassemia/Hb E cases as compared to normal controls showed 32 proteins differentially regulated between normal controls and patients and 9 proteins as differentially regulated between mild and severe cases [27]. Surprisingly, only two proteins, serum albumin and complement C3, were detected as being differentially regulated in both studies. Serum proteins altered in *β*-thalassemia/Hb E patients included proteins involved in transcriptional regulation, signal transduction, protein metabolism, and protein trafficking [27] which are perhaps reflective of the underlying pathophysiology of *β*-thalassemia/Hb E residing in ineffective erythropoiesis, while the proteins detected here (haptoglobin, haptoglobin-related protein, and apolipoprotein A-1) are much more reflective of the consequences of increased red-cell hemolysis.

In summary, this study provides a list of plasma proteins differentially expressed between normal controls and patients with HbH disease and between HbH and HbH-CS. The proteins identified may serve as good candidates for further studies on the pathophysiology of HbH disease or for biomarkers associated with severity of HbH disease.

## Figures and Tables

**Table 1:** Clinical parameters of 12 normal controls, 12 HbH, and 10 HbH-CS patients.

Parameters	Normal subjects (*n* = 12)	Patients (*n* = 22)	HbH (*n* = 12)	HbH-CS (*n* = 10)
Gender (male/female)	5/7	9/13	5/7	4/6
Hb (g/dL)∗	13.28 ± 0.97	8.74 ± 1.03	9.32 ± 1.08	8.17 ± 0.59
Hct (%)∗	39.8 ± 3.59	34.85 ± 4.08	36.43 ± 3.58	33.27 ± 4.21
WBC (×10^3^/mL)	7.02 ± 1.03	6.19 ± 1.78	6.43 ± 2.22	4.42 ± 1.37
RBC (×10^6^/mL)	4.53 ± 0.40	4.74 ± 0.98	5.02 ± 1.11	5.95 ± 0.79
MCV (fL)∗	87.78 ± 2.42	74.02 ± 5.46	73.53 ± 6.62	74.50 ± 5.75
MCH (pg)∗	29.37 ± 0.89	18.06 ± 1.78	18.03 ± 1.39	18.08 ± 2.24
MCHC (g/dL)∗	33.43 ± 0.75	24.41 ± 2.00	24.05 ± 1.30	24.32 ± 2.67

Data are presented as mean ± standard deviation (SD). Hb: hemoglobin, Hct: hematocrit, WBC: white blood cells, RBC: red blood cells, MCV: mean corpuscular volume, MCH: mean corpuscular hemoglobin, and MCHC: mean corpuscular hemoglobin concentration.

*P value <0.05 compared between normal control and HbH disease.

**Table 2:** List of 17 differential spots representing 14 different plasma proteins compared between normal control, HbH, and HbH-CS.

Number	Name of protein	MW∗	pI∗∗	MOWSE score	Sequence coverage (%)	Intensity ratio of normal control/patient	*P* value	Tissue specificity	Functions
H01	Gelsolin	86043	5.9	72	14	2.26	0.0141	Phagocytic cells, platelets, fibroblasts, muscle cells	actin-binding proteins, calcium-regulated protein

H02	Serotransferrin	79294	6.81	1180	54	1.56	0.028	Liver	Iron binding transport proteins

H03	Complement C3	79294	6.02	272	11	2.89	0.037	Liver	Phagocytosis, inflammatory responses, activation cell damage

H04	Alpha-1-antitrypsin	46878		553	37	0.72	0.044	Liver	Irreversible inhibition of trypsin

H05	Ig gamma-1 chain C region	36596	8.46	58	16	0.2	0.003	B lymphocyte	Immune response

H06	Ig gamma-2 chain C region	36505	7.66	42	16	0.38	0.015	B lymphocyte	Immune response

H10	Apolipoprotein A-I	30759	5.56	696	73	1.65	0.003	Liver	Transport of cholesterol

H11	Haptoglobin	45861	6.13	172	13	3.15	0.021	Liver	Hb-scavenging proteins, hemolytic marker
H12	Haptoglobin	45861	6.13	172	13	3.6	0.007		
H13	Haptoglobin	45861	6.13	102	13	3.64	0.013		

H14	Transthyretin	15991	5.52	325	34	3.34	0.018	Liver	Thyroxin-binding prealbumin, redox reaction

H15	Ig mu chain C region	49960	6.35	46	10	0.32	0.007	B lymphocyte	Immune response

H08	Actin	42052	5.29	141	29	4.82	0.005	Cell membrane	Red-cell skeleton membrane protein

H09	Haptoglobin-related protein	39518	5.92	115	24	3.13	0.05	Liver	Hb binding protein

H07	Serum paraoxonase	39877	5.08	72	17	4.06	0.009	Liver	Antioxidant, detoxification of organophosphates

H16	Serum albumin	71317	5.92	363	20	0.50	0.010	Liver	Regulation of the colloidal osmotic pressure of blood

H17	Serum albumin	71317	5.92	180	17	0.45	0.014

**pI: isoelectric point and ∗MW: molecular weight.

**Table 3:** List of 10 differential spots representing 7 plasma proteins compared between HbH and HbH-CS.

Number	Name of protein	MW∗	pI∗∗	MOWSE score	Sequence coverage (%)	Intensity ratio of HbH/HbH-CS	*P* value	Tissue specificity	Functions
H03	Complement C3	79294	6.02	272	11	2.96	0.077	Liver	Phagocytosis, inflammatory responses, activation cell damage

H11	Haptoglobin	45861	6.13	172	13	4.81	0.021	Liver	Hb-scavenging proteins
H12	Haptoglobin	45861	6.13	172	13	3.47	0.016		
H13	Haptoglobin	45861	6.13	102	13	2.88	0.049		

H14	Transthyretin	15991	5.52	325	34	5.48	0.002	Liver	Thyroxin-binding prealbumin, redox reaction

H08	Actin	42052	5.29	141	29	3.21	0.043	Cell membrane	Red-cell skeleton membrane protein

H09	Haptoglobin-related protein	71317	5.92	403	40	2.07	0.028	Liver	Liver damage

H07	Serum paraoxonase	39877	5.08	72	17	2.54	0.047	Liver	Antioxidant, detoxification of organophosphates

H-16	Serum albumin	71317	5.92	363	20	0.57	0.021	Liver	Regulation of the colloidal osmotic pressure of blood
H-17	Serum albumin	71317	5.92	180	17	0.49	0.039		
									

**pI: isoelectric point and ∗Mw: molecular weight.
